# A synoptic review of the genus Thaumaspis Bolívar (Orthoptera, Tettigoniidae, Meconematinae) with the description of a new genus and four new species

**DOI:** 10.3897/zookeys.443.7529

**Published:** 2014-09-29

**Authors:** Hanqiang Wang, Xianwei Liu, Kai Li

**Affiliations:** 1 School of Life Science, East China Normal University, Shanghai, 200062, China; 2 Shanghai Entomological Museum, Chinese Academy of Science, Shanghai, 200032, China

**Keywords:** Orthoptera, Tettigoniidae, Meconematinae, taxonomy, Athaumaspis, Thaumaspis, Pseudothaumaspis, new species

## Abstract

Two new species of the new genus Athaumaspis **gen. n.**, Athaumaspis minutus **sp. n.** and Athaumaspis tibetanus **sp. n.** from Vietnam and China are described. The subgenus Pseudothaumaspis of Thaumaspis is elevated to generic status and another two new species Pseudothaumaspis bispinosus **sp. n.** and Pseudothaumaspis furcocercus **sp. n.** are described, the remaining species of Thaumaspis are reviewed and keyed with the four new species.

## Introduction

The genus Thaumaspis is recognized by their opisthognathous head, short wings, and completely membranous genitalia of male. The genus was proposed by Bolívar for Thaumaspis trigonurus Bolívar, 1900. Xiphidiopsis hastaticercus Tinkham, 1936 and Xiphidiopsis yachowensis Tinkham, 1944 were placed into the genus by Bey-Bienko (1957). However, Gorochov (1993) thought Xiphidiopsis hastaticercus was similar to Chandozhinskia Gorochov, 1993 without a formal assignation. Jin and Xia (1994) listed this species in Thaumaspis. We examined specimens of this species found there was no difference between Thaumaspis hastaticercus and Chandozhinskia bivittata except for length of wings and presence of some stripes, so we believe Thaumaspis hastaticercus should be included in Chandozhinskia, as to whether they are the same species with different wing morphs or not needs molecular evidence. Xiphidiopsis yachowensis was assigned to Neocyrtopsis Liu & Zhang, 2007 by Wang et al. (2013). Afterwards, Thaumaspis was subdivided into three subgenera (Gorochov 1993, 1998) based on features of pronotum, tegmina and male genitalia: Thaumaspis s. str., Isothaumaspis Gorochov, 1993 and Pseudothaumaspis Gorochov, 1998. Subsequently, Thaumaspis (Thaumaspis) henanensis Liu & Wang, 1998 (female) and Thaumaspis (Thaumaspis) bifurcata Liu, Zhou & Bi, 2010 (male) were placed in Thaumaspis both known from singletons, but now their status seems to be short of evidence and for further study the opposite sexes are required.

Gorochov (1993, 1998) included Thaumaspis in tribe Meconematini, but the OSF website still grouped it in Meconematinae with another 31 genera out of three tribes. Actually, those genera that were excluded from tribe Phlugidini and Phisidini should be included in Meconematini. Tirbe Phlugidini is known for their very large compound eyes, both opened protibial tympana, rounded posterior margin of pronotum, shorter tegmina than wings (except brachypterous species), ventral spines of fore femora, and short also base inflated ovipositor; Phisidini is known for their stronger ventral spines of fore and middle femora, shield protibial tympana, truncated posterior margin of pronotum, long tegmina longer than or equal to wings (except brachypterous species), and longer ovipositor (some with denticulate margins at apical half); as for Meconematini, the key features are unarmed femora, opened protibial tympana at least on one side, rounded posterior margin of pronotum, tegmina no longer than wings (except brachypterous species), and longer ovipositor with smooth margins (sometimes with an apical hook). Thus according to generic characteristics, there is no doubt for inclusion of Thaumaspis in tribe Meconematini.

Thaumaspis currently contains eight species. Here we elevate the subgenus Pseudothaumaspis to normal rank in consideration of hypognathous head and unique ventral arms of male 10^th^ abdominal tergite. We also describe two new species of Pseudothaumaspis, propose a new genus Athaumaspis gen. n. which includes Thaumaspis (Thaumaspis) bifurcata Liu, Zhou & Bi, 2010 for another two new species. Four new species are from China and Vietnam: Athaumaspis minutus sp. n., Athaumaspis tibetanus sp. n., Pseudothaumaspis bispinosus sp. n. and Pseudothaumaspis furcocercus sp. n.

## Material and methods

The materials for this research were collected by us (from China) and came from the Bishop Museum (from Vietnam). Morphological structures were examined using a Leica MZ 125 and an OLYMUPS SZX 16 stereomicroscope, images were taken using a Motic Moticam Pro 252A digital imaging system, and drawings were produced by Adobe Photoshop from the digital images. All type specimens of new species are deposited in the SEM (Shanghai Entomological Museum, Chinese Academy of Science.) and the BPBM (Bernice Pauahi Bishop Museum, Hawaii).

In the specimen measurements, we measured length of body by distance between apex of fastigium verticis and posterior margin of tenth abdominal tergite, ovipositor by distance between base of subgenital fig and apex of ovipositor; pronotum, tegmina and posterfemora by distance between summit of base and apex. All length are shown in millimeter.

## Systematics

### Key to species of genus Thaumaspis Bolívar, 1900, Athaumaspis gen. n. and Pseudothaumaspis Gorochov, 1998, stat. n.

**Table:** 

1	Head hypognathous, ovipositor short and up curve, or unknown	**2**
–	Head obliquely opisthognathous, ovipositor almost straight and long	**Thaumaspis Bolívar, 1900... 7**
2	Pair of unique ventral arms at male 10^th^ abdominal tergite	**Pseudothaumaspis Gorochov, 1998, stat. n.... 3**
–	Ventral part of male 10^th^ abdominal tergite as usual	**Athaumaspis gen. n... 5**
3	Each lower lobe of hind knee with an apical spine	**4**
–	Spine of genicular lobe absent	**Pseudothaumaspis furcocercus sp. n.**
4	Apex of male cerci with 3 processes; subgential fig of female transverse	**Pseudothaumaspis gialaiensis Gorochov, 1998**
–	Male cerci robust, with 2 long inner processes; female unknown	**Pseudothaumaspis bispinosus sp. n.**
5	Pronotum of male without markings; female unknown	**6**
–	Body smaller, pronotum with blackish brown and yellow patches	**Athaumaspis minutus sp. n.**
6	Posterior marginal process on abdominal tergite 10 larger, cerci long	**Athaumaspis tibetanus sp. n.**
–	Posterior marginal process on abdominal tergite 10 very small, cerci short and stout	**Athaumaspis bifurcatus (Liu, Zhou & Bi, 2010), comb. n.**
7	Male 10^th^ abdominal tergite bearing a single process at hind margin; female ovipositor almost straight	**Thaumaspis (Thaumaspis) Bolívar, 1900... 8**
–	Male 10^th^ abdominal tergite without processes at hind margin; female unknown	**Thaumaspis (Isothaumaspis) Gorochov, 1993** **Thaumaspis (Isothaumaspis) forcipatus Bolívar, 1900**
8	Female subgenital fig almost triangular	**9**
–	Female subgenital fig nearly quadrate, hind margin circularly truncate; male unknown	**Thaumaspis (Thaumaspis) longipes Bolívar, 1900**
9	Single process of male 10^th^ abdominal tergite triangular, apex sparsely denticulated, male cerci with distinct processes; female subgenital fig not transverse, apex sharp	**Thaumaspis (Thaumaspis) trigonurus Bolívar, 1900**
–	Single process of male 10^th^ abdominal tergite longer, separate into 2 lobes apically, male cerci without process; female subgenital fig more or less transverse, apex blunt	**10**
10	Middle process of male 10^th^ abdominal tergite rearwards produced, male cerci extremely bent inwards; female subgenital fig hardly transverse, nearly circular	**Thaumaspis? siccifolii (Karny, 1922)**
–	Male unknown; female subgenital fig transverse, circular or triangular	**11**
11	Fastigium of vertex conical; female subgenital fig nearly triangular	**Thaumaspis (Thaumaspis) montanus Bey-Bienko, 1957**
–	Fastigium of vertex cylindrical; female subgenital fig nearly circular	**Thaumaspis (Thaumaspis) castetsi Gorochov, 1993**

#### Genus Thaumaspis

Taxon classificationAnimaliaOrthopteraTettigoniidae

Bolívar, 1900

Thaumaspis : Bolívar 1900: 768, t. 11, figs 11a–b; Kirby 1906: 373; Caudell 1912: 2; Karny 1924: 135; Beier 1966: 280; Gorochov 1993: 261; Jin and Xia 1994: 26; Otte 1997: 97; Gorochov 1998: 114.

##### Type species.

Thaumaspis trigonurus Bolívar, 1900.

##### Description.

Body small sized. Head opisthognathous. Fastigium of vertex short without sulcus dorsally, face extremely oblique, last segment of maxillary palpi longer than the preceding. Pronotum with low lateral lobes, humeral sinus absent; auditory foramina of thorax entirely exposed. Tegmina shorter than pronotum, with the stridulating organ in male, hind wing degraded. Auditory foramina of fore tibiae opened, hind tibiae with 2 pairs of apical spurs. Male 10^th^ abdominal tergite bearing a single process on posterior margin or absence, cerci elongate with processes, subgenital fig with short styli, genitalia entirely membranous. Female subgenital fig nearly triangular, ovipositor shorter than hind femora, ventral valve with a small apical hook.

##### Diagnosis.

The opisthognathous head and low lateral lobes of pronotum can easily distinguished them from other genera, for now Thaumaspis is the only genus with opisthognathous head of tribe Meconematini.

#### Subgenus Thaumaspis

Taxon classificationAnimaliaOrthopteraTettigoniidae

Gorochov, 1993

Thaumaspis (Thaumaspis) : Gorochov 1993: 261; Jin and Xia 1994: 26; Otte 1997: 97; Gorochov 1998: 114.

##### Type species.

Thaumaspis trigonurus Bolívar, 1900.

##### Diagnosis.

Pronotum shorter, tegmina extremely short and truncate at apex, male 10^th^ abdominal tergite attached a single process at hind margin and female subgenital fig nearly triangular.

#### 1. Thaumaspis (Thaumaspis) trigonurus

Taxon classificationAnimaliaOrthopteraTettigoniidae

Bolívar, 1900

Figs 1–6

Thaumaspis trigonurus : Bolívar 1900: figs 11, a– b; Kirby 1906: 373; Caudell 1912: 3; Beier 1966: 280.
Thaumaspis (Thaumaspis) trigonurus : Gorochov 1993: figs 169– 176, 261– 262; Gorochov 1998: 114.

##### Diagnosis.

Apex of the male posterior process at 10 abdominal tergite sparsely dentate (Fig. 2). Cerci slightly curved, basal half with numerous short processes (Figs 3, 4), styli short. Female subgenital fig triangular, apex slightly sharp (Fig. 5).

##### Coloration.

Body greenish, unicolor.

##### Measurement.

(length in mm) Body, ♂♀9.0; pronotum, ♂♀3.8; tegmina, ♂♀0.5–1.5; hind femora, ♂♀7.0; ovipositor, ♀7.0.

##### Distribution.

India.

**Figures 1–6.**
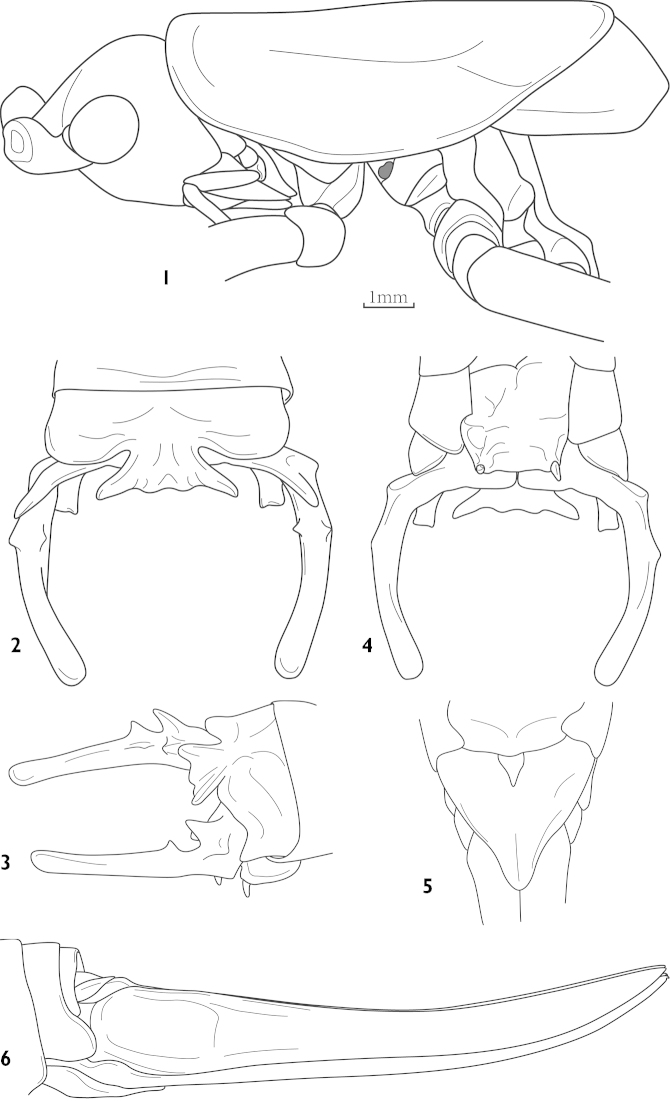
Thaumaspis (Thaumaspis) trigonurus Bolívar, 1900 (after OSF website) **1** head, pronotum and tegmina, lateral view **2** end of male abdomen, dorsal view **3** end of male abdomen, lateral view **4** end of male abdomen, ventral view **5** subgenital fig of female, ventral view **6** female abdomen terminal, lateral view.

#### 2. Thaumaspis? siccifolii

Taxon classificationAnimaliaOrthopteraTettigoniidae

(Karny, 1922)

Figs 7–11

Cecidophaga siccifolii : Karny 1922: 299, fig. 3.
Thaumaspis siccifolii : Karny 1924: 135, figs 54 a– f; Beier 1966: 280.
Thaumaspis (Thaumaspis) siccifolii : Otte 1997: 98.

##### Diagnosis.

Head hypognathous. Male 10^th^ abdominal tergite transverse, middle lobe divided into 2 finger-shaped apices (Fig. 8). Cerci extremely incurved, ventral base and subapex widened (Fig. 9). Subgenital fig almost trapezoidal. Female subgenital fig almost circular (Fig. 10).

##### Coloration.

Body olive-green, eyes darkish, antennae with dark rings.

##### Measurement.

(length in mm) Body, ♂8.0, ♀8.5–9.5; pronotum, ♂3.0, ♀2.5; tegmina, ♂♀3.0; hind femora, ♂♀8.0–8.5; ovipositor, ♀5.5–6.0.

##### Discussion.

The general features of this species ally to Cecidophagula, such as characters of head and wings. Actually, it had been described as a Cecidophagula originally before Karny assigned it to Thaumaspis in consideration of the single process of genital segments. Gorochov excluded this species in his study (1993, 1998). Since we are unable to examine type material we still leave it in Thaumaspis.

##### Distribution.

Indonesia.

**Figures 7–11.**
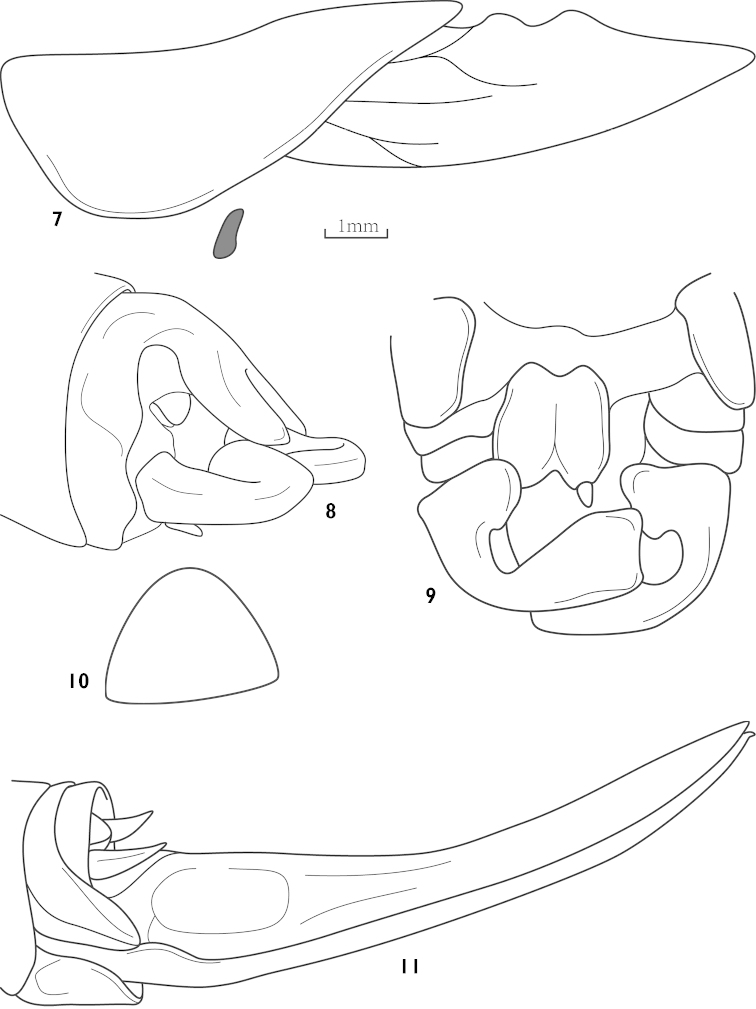
Thaumaspis? *siccifolii* (Karny, 1922) (after OSF website and Karny) **7** pronotum and tegmina, lateral view **8** end of male abdomen, dorsal-lateral view **9** end of male abdomen, ventral view **10** subgenital fig of female, ventral view (after Karny) **11** female abdomen terminal, lateral view.

#### 3. Thaumaspis (Thaumaspis) montanus

Taxon classificationAnimaliaOrthopteraTettigoniidae

Bey-Bienko, 1957

Figs 12–13

Thaumaspis montanus : Bey-Bienko 1957: 411, fig. 13; Bey-Bienko 1962: 135; Beier 1966: 280; Liu and Jin1994: 109; Jin and Xia1994: 26.
Thaumaspis (Thaumaspis) montana : Gorochov 1993: 82; Gorochov 1998: 114, figs 102– 103.

##### Diagnosis.

Female tegmina rather shorter than pronotum, subgenital fig short, nearly triangular, apex blunt (Fig. 13). Male unknown.

##### Coloration.

Body yellowish (maybe greenish in life), totally unicolor.

##### Measurement.

(length in mm) Body, ♀9.5; pronotum, ♀3.7; tegmina, ♀3.2; hind femora, ♀8.5; ovipositor, ♀7.5.

##### Distribution.

China (Yunnan, Tengchong).

**Figures 12–13.**
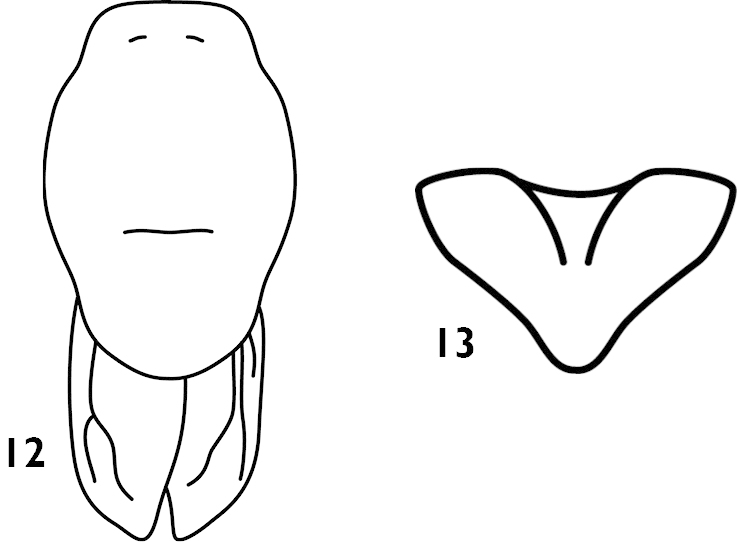
Thaumaspis (Thaumaspis) montana Bey-Bienko, 1957 (after Gorochov) **12** pronotum and tegmina, dorsal view **13** subgenital fig of female, ventral view.

#### 4. Thaumaspis (Thaumaspis) castetsi

Taxon classificationAnimaliaOrthopteraTettigoniidae

Gorochov, 1993

Figs 14 –16

Thaumaspis (Thaumaspis) castetsi : Gorochov 1993: 83, figs 177– 179, 263; Gorochov 1998: 114.

##### Diagnosis.

Female subgenital fig transverse, hind margin with middle circular convex (Fig. 15). Ovipositor almost straight (Fig. 16). Male unknown.

##### Coloration.

Body yellowish, unicolor.

##### Measurement.

(length in mm) Body, ♀11.0; pronotum, ♀3.7; tegmina, ♀2.4; hind femora, ♀7.5; ovipositor, ♀7.0.

##### Distribution.

India.

**Figures 14–16.**
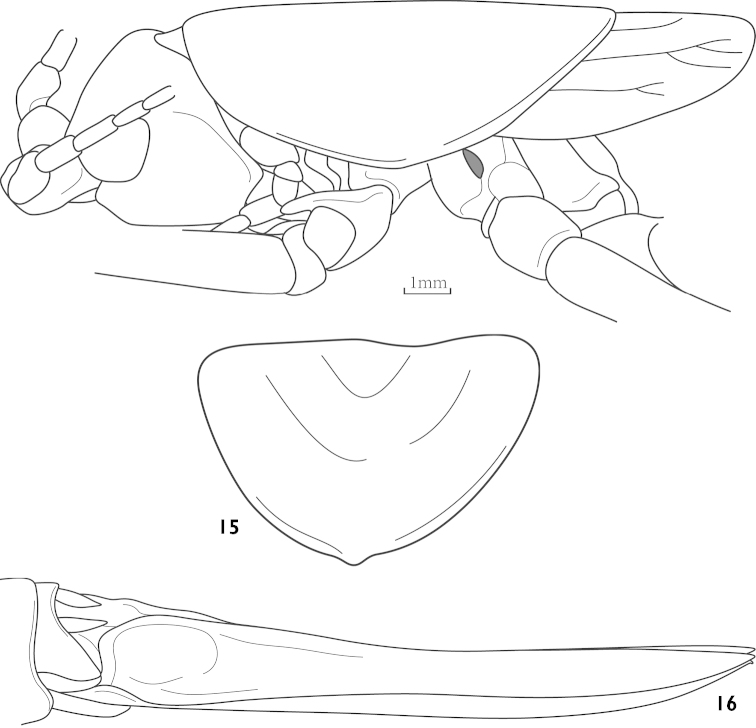
Thaumaspis (Thaumaspis) castetsi Gorochov, 1993 (after OSF website) **14** head, pronotum and tegmina, lateral view **15** subgenital fig of female, ventral view **16** female abdomen terminal, lateral view.

#### 5. Thaumaspis (Thaumaspis) longipes

Taxon classificationAnimaliaOrthopteraTettigoniidae

Bolívar, 1900

Figs 17 –19

Thaumaspis longipes : Bolívar 1900: 769, t. 11, fig. 13; Kirby 1906: 373; Caudell 1912: 3; Beier 1966: 280.
Thaumaspis (*subgenus*?) *longipes*: Gorochov 1993: 83, figs 184– 186, 265.
Thaumaspis (Pseudothaumaspis?) *longipes*: Gorochov 1998: 114– 115.

##### Diagnosis.

Female tegmina no longer than pronotum, apex pointed. Subgenital fig nearly quadrate, hind margin circularly truncated (Fig. 19). Male unknown.

##### Coloration.

Body greenish, unicolor.

##### Measurement.

(length in mm) Body, ♀12.0; pronotum, ♀3.8; tegmina, ♀3.0; hind femora, ♀10.0; ovipositor, ♀10.0.

##### Distribution.

India (New Delhi).

**Figures 17–19.**
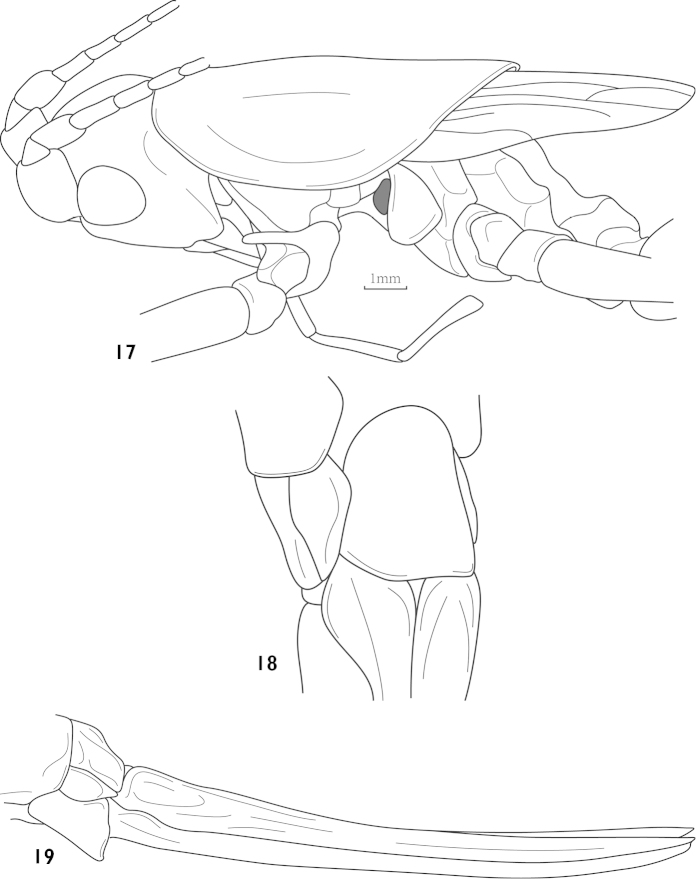
Thaumaspis (Thaumaspis) longipes Bolívar, 1900 (after OSF website) **17** pronotum and tegmina, lateral view **18** subgenital fig of female, ventral view **19** female abdomen terminal, lateral view.

#### Subgenus Isothaumaspis

Taxon classificationAnimaliaOrthopteraTettigoniidae

Gorochov, 1993

Thaumaspis (Isothaumaspis) : Gorochov 1993: 83, figs 181–183.

##### Type species.

Thaumaspis forcipatus Bolívar, 1900.

**Diagnosis.** This subgenus differs from nominotypical subgenus by longer pronotum equal to tegmina, posterior marginal processes of male 10^th^ abdominal tergite absent.

#### 6. Thaumaspis (Isothaumaspis) forcipatus

Taxon classificationAnimaliaOrthopteraTettigoniidae

Bolívar, 1900

Figs 20 –23

Thaumaspis forcipatus : Bolívar 1900: 769, t. 11, figs 12a–b; Kirby 1906: 373; Caudell 1912: 3; Beier 1966: 280.
Thaumaspis (Isothaumaspis) forcipatus : Gorochov 1993: 83, figs 180– 183, 264; Gorochov 1998: 114.

##### Diagnosis.

**Figures 20–23.**
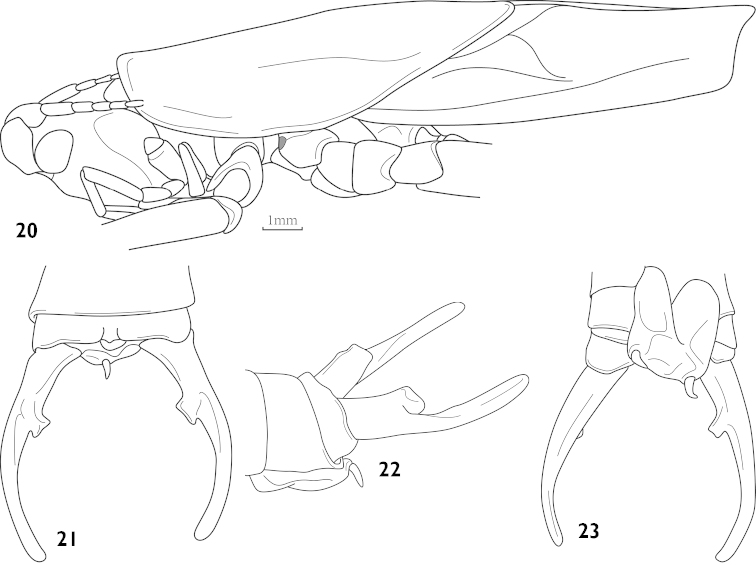
Thaumaspis (Isothaumaspis) forcipatus Bolívar, 1900 (after OSF website) **20** head, pronotum and tegmina, lateral view **21** end of male abdomen, dorsal view **22** end of male abdomen, lateral view **23** end of male abdomen, ventral view.

Male pronotum longer, tegmina almost equal to pronotum, apex truncate (Fig. 20). 10^th^ abdominal tergite with a median notch at middle of hind margin (Fig. 21). Cerci longer, slightly incurved, median portion with one lobe truncated in apex (Fig. 22). Female unknown.

##### Measurement.

(length in mm) Body, ♂11.0; pronotum, ♂4.8; tegmina, ♂4.8; hind femora, ♂5.5.

##### Distribution.

India.

#### Genus Athaumaspis

Taxon classificationAnimaliaOrthopteraTettigoniidae

Wang & Liu gen. n.

http://zoobank.org/D0F10EB4-C1F6-440C-A6B0-6723D1BD0506

##### Type species.

Athaumaspis minutus sp. n.

##### Description.

Body small of this tribe. Head hypognathous, low in profile. Fastigium of vertex short with shallow furrow dorsally, last segment of maxillary palpi little longer than the preceding. Pronotum with lower paranota, humeral sinus absent; auditory foramina of thorax entirely exposed. Tegmina shorter than pronotum, with the stridulating organ in male, hind wing degraded. Auditory foramina of fore tibiae opened, hind tibiae with 2 pairs of apical spurs. Male 10^th^ abdominal tergite with branched process at posterior margin, cerci elongate or branched, subgenital fig with short styli, genitalia entirely membranous. Female subgenital fig transverse, rounded at posterior margin, ovipositor short and upcurved, ventral valve with a small apical hook.

##### Diagnosis.

This new genus similar to Thaumaspis in body size and bearing posterior marginal process of abdominal tergite 10, but quite different by hypognathous head, marginal process of abdominal tergite 10 bifurcated.

#### 7. Athaumaspis minutus

Taxon classificationAnimaliaOrthopteraTettigoniidae

Wang & Liu sp. n.

http://zoobank.org/829CEB3D-6C75-4098-8773-C9BBE472BF5C

Figs 24 –30

##### Materials.

Holotype♂, paratype2♀♀, Vietnam, Mt. Lang Bian, Alt. 1500– 2000m, 1961.V.19– VI.8, coll. N.R. Spencer (BPBM). Deposited in SEM temporarily.

##### Description.

**Figures 24–30.**
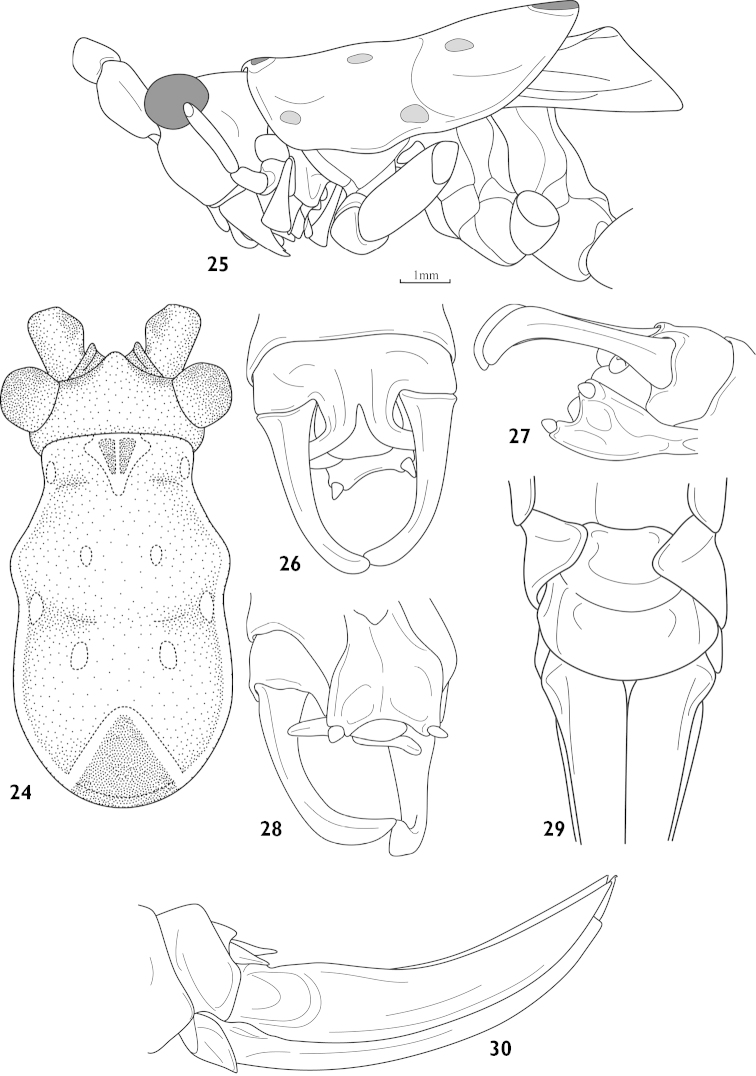
Athaumaspis minutus sp. n. **24** head, pronotum and tegmina, dorsal view **25** head, pronotum and tegmina, lateral view **26** end of male abdomen, dorsal view **27** end of male abdomen, lateral view **28** end of male abdomen, ventral view **29** subgenital fig of female, ventral view **30** female abdomen terminal, lateral view.

Male. Head low in profile. Fastigium of vertex rather short, shallowly furrowed on dorsum (Fig. 24), face slightly oblique (Fig. 25), compound eyes oval and protruded outwards, last segment of maxillary palpi longer than preceding. The superior and inferior edge of pronotum nearly paralled from a lateral view, metazona slightly elevated, paranota of pronotum lower, hind margin rounded, humeral sinus absent; auditory foramina of thorax entirely exposed. Tegmina shorter than pronotum, hind margin obliquely truncated, hind wings reduced. Fore tibiae armed spines of type 4, 4 (1, 1) on either margin of ventral surface, hind tibiae with 20– 23 dorsal teeth each margin above and 2 pairs of apical spurs. Posterior margin of abdominal tergite 10 with middle process, distinctly branched (Fig. 26). Epiproct reduced. Cerci elongate, rather simple, incurved in its apical third, dorsal surface with weak keels (Fig. 27). Subgenital fig narrowed basally, broad in apical half, hind margin roundly emarginate with rather short styli (Fig. 28).

Female. General roughly as in male. Cerci short and conical, subgenital fig transverse and flabellate, hind margin circular convex (Fig. 29). Ovipositor is short, upcurved, ventral valve with a weak apical hook.

##### Coloration.

Body yellowish (maybe greenish in life), eyes blackish brown, antennae with inconspicuous darkish rings, fore and hind margins of pronotum either with blackish brown marking rounded yellow rim (Fig. 24), lateral lobe with 4 yellow markings.

##### Measurement.

(length in mm) Body, ♂7.5, ♀8.0; pronotum, ♂2.8, ♀2.2; tegmina, ♂1.0, ♀0.8; hind femora, ♂6.5, ♀7.0; ovipositor, ♀3.8.

##### Diagnosis.

This new species distinguishes from other species of the genus in body smaller, pronotum with blackish brown and yellow markings, female subgential fig with rounded posterior edge.

##### Etymology.

The specific epithet referrers body form of this species, from Latin minūtus. The gender of the epithet is masculine.

##### Distribution.

Vietnam.

#### 8. Athaumaspis tibetanus

Taxon classificationAnimaliaOrthopteraTettigoniidae

Wang & Liu sp. n.

http://zoobank.org/C0DE31AE-E56B-47E3-AC1E-5141CBAB73BA

Figs 31 –34

##### Materials.

Holotype ♂ (# 14088760), China, Xizang, Nyalam Country, Zhangmu, Alt. 2300m, 2010.VII.17–18, coll. W.X. Bi; Paratype 1♂ (# 14088761), same data as holotype (SEM).

##### Description.

**Figures 31–34.**
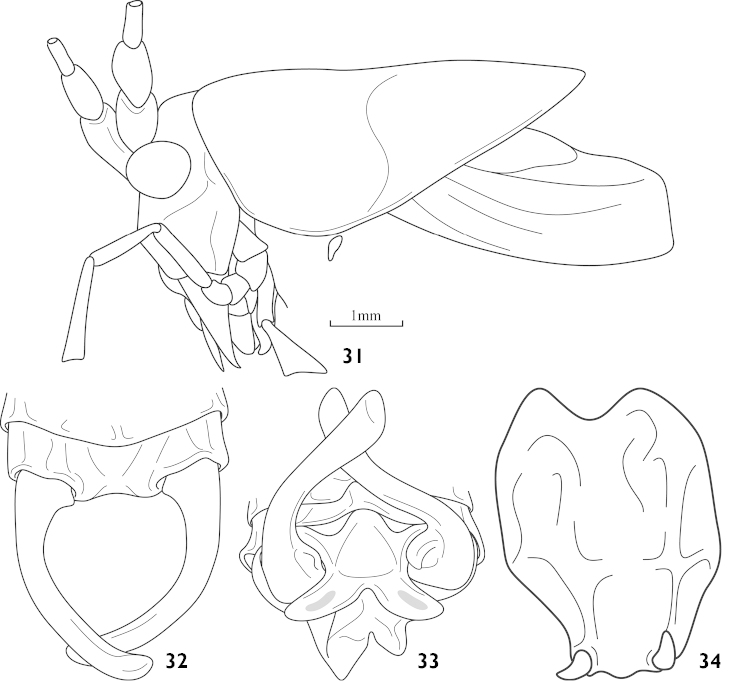
Athaumaspis tibetanus sp. n. based on # 14088761 (**31, 34**) and # 14088760 (**32, 33**) **31** head, pronotum and tegmina, lateral view **32** end of male abdomen, dorsal view **33** middle process of male 10^th^ abdominal tergite, rear view **34** subgenital fig of male, ventral view.

Male. Head high in profile. Fastigium of vertex short, dorsum shallowly furrowed in middle, face slightly oblique (Fig. 31), but higher, compound eyes subovoid and moderately protruded, last segment of maxillary palpi slightly longer than preceding. Protonum nearly triangular in profile, metazona little elevated, paranota higher, hind margin obliquely truncated without humeral sinus; auditory foramina of thorax entirely exposed. Tegmina shorter than pronotum by one third, posterior edge truncated; hind wings deduced. Fore tibiae spines armed 4, 4 (1, 1), hind tibiae with 19–20 dorsal teeth either margin above and 2 pairs of apical spurs. 10^th^ abdominal tergite bearing an extended process at the middle of hind margin, bending vertically downwards and invisible dorsally (Fig. 32), apex distinctly branched (Fig. 33). Epiproct reduced. Cerci longer, inner surface of base occurs a lobe, incurved in one third, apex moderately expanding. Subgenital fig longer than width, apical two fifth narrowing towards tip, little convex at median hind margin, styli short (Fig. 34).

Female unknown.

##### Coloration.

Body yellowish (may be greenish in life), unicolor.

##### Measurement.

(length in mm) Body, ♂7.0–8.0; pronotum, ♂3.3–3.5; tegmina, ♂2.0; hind femora, ♂6.5–7.0.

##### Diagnosis.

This species looks different from type species in general, distinguishes mainly by higher head and pronotum in profile and inner lobe of cerci; but shearing branched process of male 10th abdominal tergite and simple but slender cerci.

##### Etymology.

The specific epithet is Latinized name of district Tibet where this species distributed. The gender of the epithet is masculine.

##### Distribution.

China (Xizang).

#### 9. Athaumaspis bifurcatus

Taxon classificationAnimaliaOrthopteraTettigoniidae

(Liu, Zhou & Bi, 2010) comb. n.

Figs 35 –36

Thaumaspis bifurcata : Liu et al. 2010: 81.

##### Diagnosis.

Posterior margin of male 10^th^ abdominal tergite with a small branched process, male cercus stout and bifurcated, superior ramus clubbed, inferior ramus with an inner triangular lobe at proximal part.

##### Coloration.

Body yellowish green. Eyes and spines of hind tibiae blackish, genicular lobes of all femora each with a blackish spot.

##### Material examined.

Holotype♂, paratype1♂, Daitianping, Fengyangshan National Nature Reserve, Zhejiang, Alt. 1200m, 2008.X.20, coll. S.L. Liu.

##### Measurement.

(length in mm) Body, ♀6,5; pronotum, ♀3.5; tegmina, ♀2.0; hind femora, ♀6.5.

##### Discussion.

The cerci of this species are stouter and quite different from previous 2 species of this genus which makes it easy to tell them apart, meanwhile the species meets the diagnosis of Athaumaspis in hypognathous head and bifurcated posterior marginal process of abdominal tergite 10. According to the features of male here we treat this species as an Athaumaspis. The specific epithet of this species originally was feminine, primarily based on Gorochov (1993), but according to type species of Thaumaspis the genus is masculine and ‘bifurcata’ should be ‘bifurcatus’, The same apply for Athaumaspis and Pseudothaumaspis.

##### Distribution.

China (Zhejiang).

**Figures 35–36.**
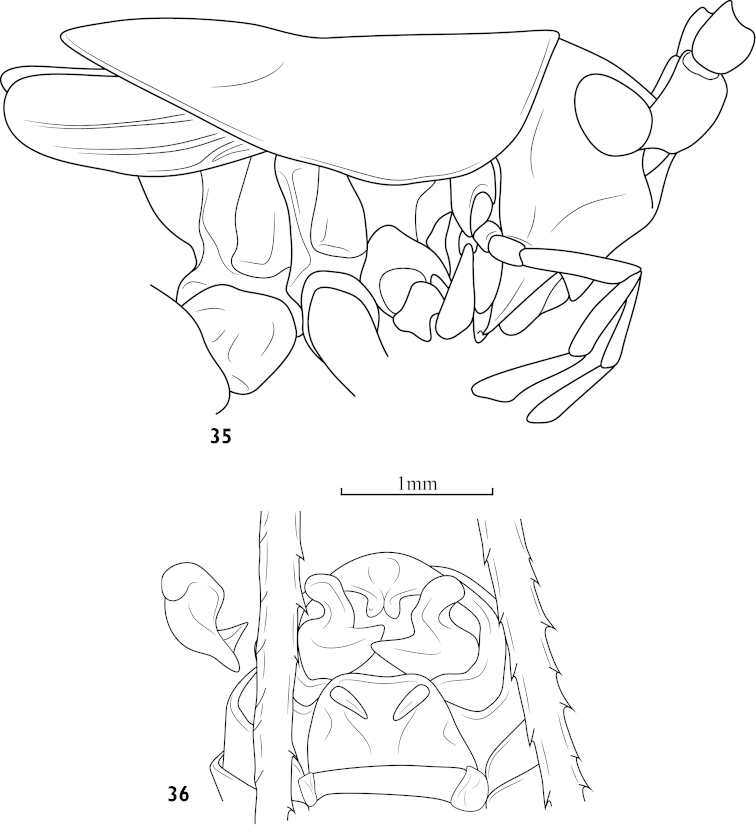
Athaumaspis bifurcatus (Liu, Zhou & Bi, 2010) comb. n. **35** head, pronotum and tegmina, lateral view **36** end of male abdomen, ventral-rear view, and right cercus, dorsal view.

#### Genus Pseudothaumaspis

Taxon classificationAnimaliaOrthopteraTettigoniidae

Gorochov, 1998 stat. n.

Thaumaspis (Pseudothaumaspis) : Gorochov 1998: 115.

##### Type species.

Pseudothaumaspis gialaiensis Gorochov, 1998.

##### Diagnosis.

This genus differs from Thaumaspis by hypognathous head, differs form all previous genera by unusual ventral arms at lower part of male 10^th^ abdominal tergite, moreover posterior marginal processes of this tergite absence or instead of by small lobes, varied apex of cerci and almost trilobed posterior part of female subgenital fig. We believed hypognathous head and unique ventral arm of male 10^th^ abdominal tergite sufficient to exclude Pseudothaumaspis from Thaumaspis and elevate it to generic status.

#### 10. Pseudothaumaspis gialaiensis

Taxon classificationAnimaliaOrthopteraTettigoniidae

Gorochov, 1998

Figs 37 –41

Thaumaspis (Pseudothaumaspis) gialaiensis : Gorochov 1998: 115, figs 104– 109.

##### Diagnosis.

**Figures 37–41.**
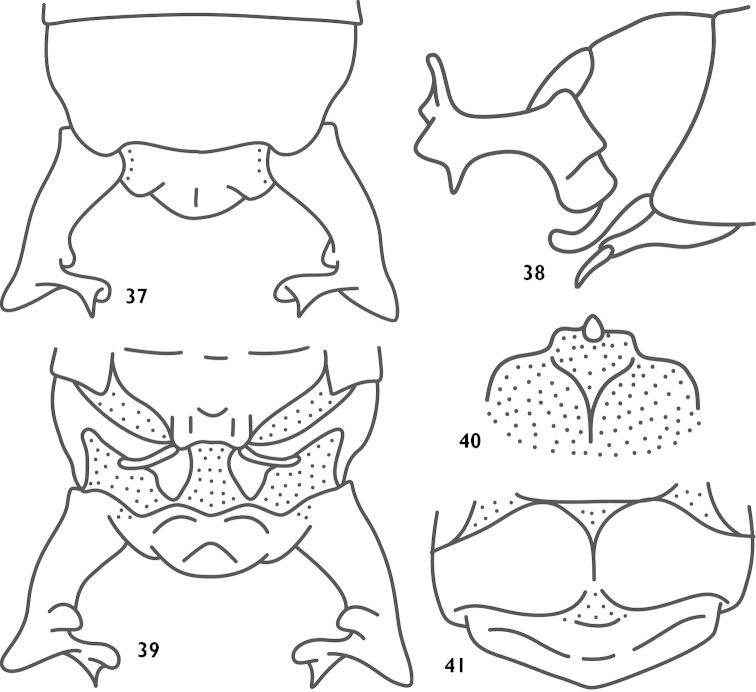
Pseudothaumaspis gialaiensis Gorochov, 1998 (after Gorochov) **37** end of male abdomen, dorsal view **38** end of male abdomen, lateral view **39** end of male abdomen, ventral view **40** male genitals, dorsal view **41** subgenital fig of female, ventral view.

Lower lobe of the male hind knee with spine, apex of male cerci with 3 processes (Figs 37–39), shorter subgenital fig with longer styli, genital smaller, apex with small a sclerous corium (Fig. 40). Female subgential fig transverse (Fig. 41), hind margin bent downwards; ventral valve of ovipositor with an apical hook.

##### Coloration.

Body yellowish green, almost unicolor, antennae with brown rings, lower part of the pronotum lateral lobe with brown edge; apex of tibiae, tarsus and spine of tibiae darkened.

##### Measurement.

(length in mm) Body, ♂13.0– 14.0, ♀12.0– 13.0; pronotum, ♂4.2– 4.5, ♀4.0– 4.2; tegmina, ♂4.0– 4.5, ♀3.0; hind femora, ♂13.5– 14.0, ♀14.0– 15.0; ovipositor, ♀5.8– 6.0.

##### Distribution.

Vietnam.

#### 11. Pseudothaumaspis bispinosus

Taxon classificationAnimaliaOrthopteraTettigoniidae

Wang & Liu sp. n.

http://zoobank.org/C3EBFD67-A6E6-4B04-9A2D-243437D35BB6

Figs 42 –45

##### Materials.

Holotype♂, Vietnam, 40km S of Dilanh (Djiring), Alt. 543m, 1960.IV.26, coll. L.W. Quate (BPBM). Deposited in SEM temporarily.

##### Description.

**Figures 42–45.**
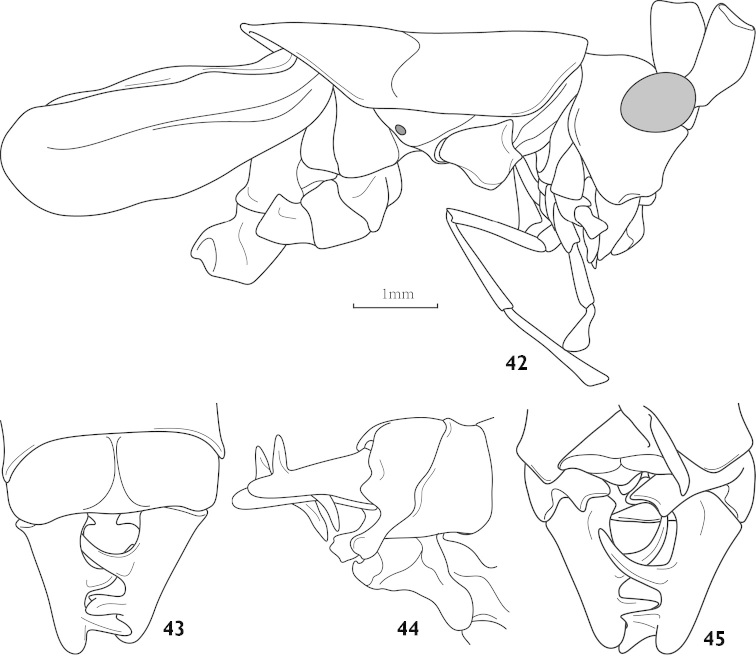
Pseudothaumaspis bispinosus sp. n. **42** head, pronotum and tegmina, lateral view **43** end of male abdomen, dorsal view **44** end of male abdomen, lateral view **45** end of male abdomen, ventral view.

Male. Fastigium of vertex short, without dorsal groove, face slightly oblique (Fig. 42), compound eyes oval and protruded forwards and outwards, last segment of maxillary palpi slightly longer than preceding. The superior edge and inferior edge of pronotum paralleled and almost straight, paranota lower, front margin little sinuate, hind margin straight without humeral sinus and obliquely truncated; auditory foramina of thorax small and entirely exposed. Tegmina almost equal to pronotum, apex rounded; hind wings degenerate. Fore tibiae armed ventral spines of type 4, 4 (1, 1), lower lobe of the hind knee bearing a spine, hind tibiae with 28–31 dorsal teeth each margin above and 2 pairs of apical spurs. Hind margin of 10^th^ abdominal tergite little sinuate (Fig. 43), lower part becoming a pair of elongate branches (Figs 44–45); cerci robust, generally conical and apex blunt, but each with 2 long inner processes: prior one downward, posterior one upward and little branched at apex. Subgenital fig damaged.

Female unknown.

##### Coloration.

Body yellowish (may be greenish alive), eyes blackish brown, antennae with inconspicuous darkish rings.

##### Measurement.

(length in mm) Body, ♂12.0; pronotum, ♂3.8; tegmina, ♂4.0; hind femora, ♂12.0.

##### Discussion.

This new species is similar to Pseudothaumaspis gialaiensis Gorochov, 1998, but differs mainly in the appearance of the male cerci. The single specimen of this species is in bad condition, especially the abdomen. Fortunately, the unique ventral arms of the 10^th^ abdominal segment and the cerci are intact.

##### Etymology.

The specific epithet refers to the character of male cerci which bearing 2 spinous processes, compose by prefix 'bi-' which means double and 'spinosus' which means spiny.

##### Distribution.

Vietnam.

#### 12. Pseudothaumaspis furcocercus

Taxon classificationAnimaliaOrthopteraTettigoniidae

Wang & Liu sp. n.

http://zoobank.org/31992FC9-40FC-4628-871E-2B60DC7E1464

Figs 46 –55

##### Materials.

Holotype♂ (# 14086640), China, Guangxi, Wuming, Damingshan, Alt. 1250m, 2013.VII.19–25, coll. W.B. Zhu, X.W. Liu, H.Q. Wang, H.G. Zhang. Paratype1♂ (# 14088762) 1♀ (# 14088763), same data as holotype (SEM).

##### Description.

**Figures 46–47.**
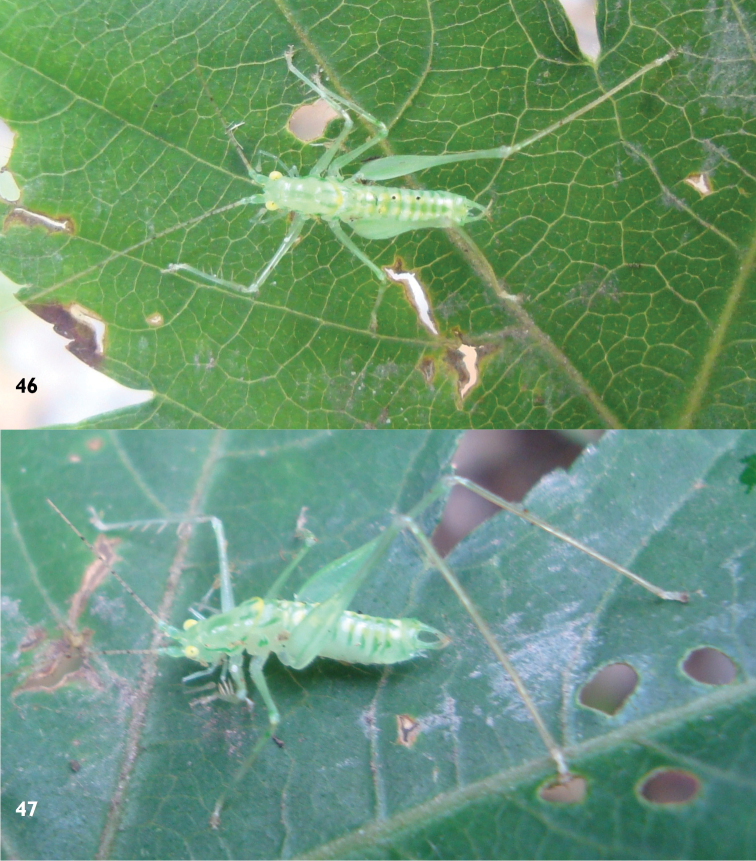
Pseudothaumaspis furcocercus sp. n., ecological photograph, lateral view and dorsal view.

**Figures 48–55.**
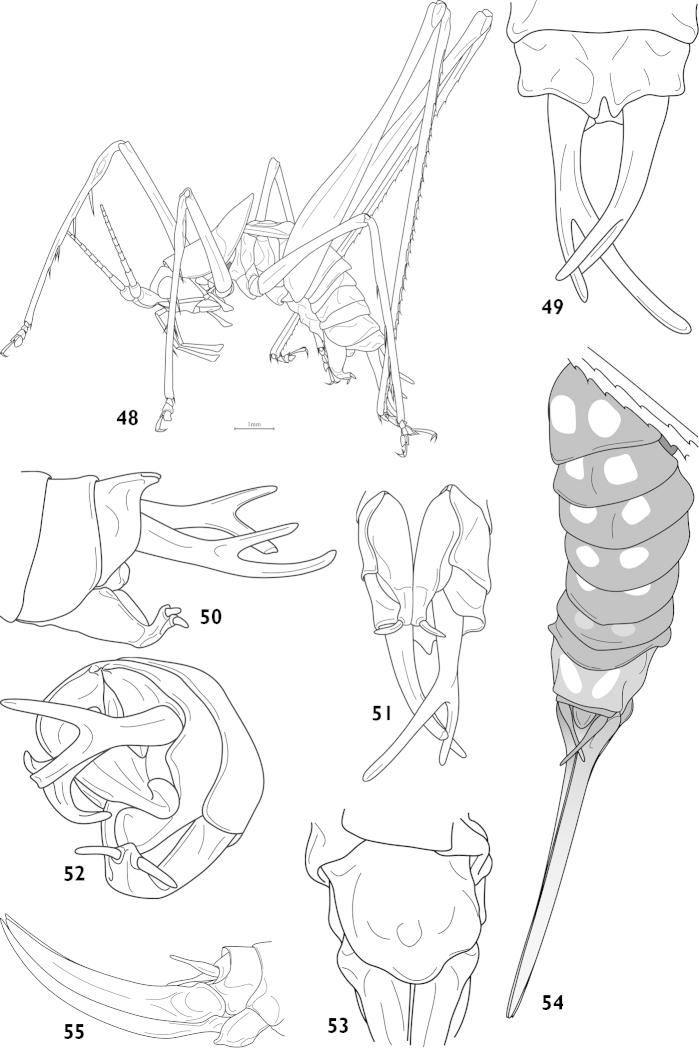
Pseudothaumaspis furcocercus sp. n. based on # 14086640 (**48–52**) and # 14088763 (**52–55**) **48** male body, lateral view **49** end of male abdomen, dorsal view (left cerci damaged at apex) **50** end of male abdomen, lateral view **51** end of male abdomen, ventral view **52** end of male abdomen, rear view **53** female subgenital fig, ventral view **54** female abdomen (2–11segments), dorsal view **55** ovipositor, lateral view.

Male. Body form small and slender. Fastigium of vertex short, without dorsal groove, face slightly oblique (Fig. 48), compound eyes subglobular, last segment of maxillary palpi slightly longer than preceding. Pronotum saddle shaped in lateral view, paranota with concave dorsal margins, ventral margin rounded, humeral sinus absent, transverse sulcus distinct especially posterior one, metazona short, rather pointed at the posterior tip; auditory foramina of thorax small and exposed. Tegmina shoter than pronotum by one third, apex truncate; hind wings degenerate. Legs very long and rather thin (Fig. 46), fore tibiae with ventral spines armed 4, 4 (1, 1), lower lobe of the hind knee without spine, hind tibiae with 21– 29 dorsal teeth either margin above and 2 pairs of apical spurs. Posterior median edge of 10^th^ abdominal tergite elongate with a deep apical incision forming 2 small lobes (Fig. 49), lower area bearing a pair of ventral arms, not elongate and apex oblate (Fig. 52). Slim cerci branched at a half, incurved, lower branch longer. Subgenital base broad, apical one third narrow and up curved, styli very long (Figs 50–52).

Female. Body form similar to that of male. Fastigium of vertex little longer and more slender, a shallow furrow on the dorsum, last segment of maxillary palpi much longer than preceding. Paranota of pronotum subacute at inferior margin, transverse sulcus distinct as in male, but metazona even shorter. Tegmina short as in male, pointed at apex, inferior margin obliquely truncated, veins conspicuous. Posterior margin of 9^th^ abdominal tergite straight, cercus slender, fusiform, apex thin and acute (Fig. 54); subgenital fig downward swell, trilobed at hind margin, mesolobe prominently convex. Ovipositor short, base upcurved, ventral valve without apical hook.

##### Coloration.

In life of male. Body lightish green, emerald green and lightish yellow variegated. Flagella pale brown with darkish rings, scape and pedicel consistent with body color. Compound eyes vivid yellow. Both lateral rims of pronotum emerald green, but posterior edge vivid yellow, dorsum with green longitudinal stripes and patches. Each abdomen tergite with a pair of bright yellow oval patches and posterior edge darkish green. Hind tibiae, Tarsi and cerci terminal pale brown.

Dry specimen. Body brownish, antennae with inconspicuous darks rings, forma and tibia darkened around the knee joint. Male unicolor; female abdomen largely blackish brown, ventral surface totally black including subgenital fig, abdomen tergites each compact with a pair of large pale patches dorsally, base of ovipositor darkened.

##### Measurement.

(length in mm) Body, ♂7.4–8.7, ♀10.2; pronotum, ♂3.2–3.6, ♀3.8; tegmina, ♂1.9, ♀1.5; hind femora, ♂8.3–8.9, ♀9.5; ovipositor, ♀4.5.

##### Etymology.

The specific epithet from Latin ‘forca’ + ‘cercus’, corresponding the feature of male bifurcate cerci. The gender of the epithet is masculine.

##### Discussion.

Bearing those unique arms, clearly it is a Pseudothaumaspis, but tegmina terminal, lower lobe of hind knee, small lobes of posterior edge of last abdominal tergite are quite different from previous species.

##### Distribution.

China (Guangxi).

## Supplementary Material

XML Treatment for Thaumaspis

XML Treatment for Thaumaspis

XML Treatment for Thaumaspis (Thaumaspis) trigonurus

XML Treatment for Thaumaspis? siccifolii

XML Treatment for Thaumaspis (Thaumaspis) montanus

XML Treatment for Thaumaspis (Thaumaspis) castetsi

XML Treatment for Thaumaspis (Thaumaspis) longipes

XML Treatment for Isothaumaspis

XML Treatment for Thaumaspis (Isothaumaspis) forcipatus

XML Treatment for Athaumaspis

XML Treatment for Athaumaspis minutus

XML Treatment for Athaumaspis tibetanus

XML Treatment for Athaumaspis bifurcatus

XML Treatment for Pseudothaumaspis

XML Treatment for Pseudothaumaspis gialaiensis

XML Treatment for Pseudothaumaspis bispinosus

XML Treatment for Pseudothaumaspis furcocercus
